# Putative presynaptic dopamine dysregulation in schizophrenia is supported by molecular evidence from post-mortem human midbrain

**DOI:** 10.1038/tp.2016.257

**Published:** 2017-01-17

**Authors:** T D Purves-Tyson, S J Owens, D A Rothmond, G M Halliday, K L Double, J Stevens, T McCrossin, C Shannon Weickert

**Affiliations:** 1 Schizophrenia Research Institute, Sydney, NSW, Australia; 2 Schizophrenia Research Laboratory, Neuroscience Research Australia, Sydney, NSW, Australia; 3 School of Psychiatry, University of New South Wales, Sydney, NSW, Australia; 4 Ageing and Neurodegeneration, Neuroscience Research Australia, Sydney, NSW, Australia; 5 School of Medical Sciences, University of New South Wales, Sydney, NSW, Australia; 6 Discipline of Biomedical Science and Brain and Mind Centre, School of Medical Sciences, Sydney Medical School, University of Sydney, Sydney, NSW, Australia; 7 New South Wales Brain Tissue Resource Centre, Discipline of Pathology, Sydney Medical School, University of Sydney, Sydney, NSW, Australia

## Abstract

The dopamine hypothesis of schizophrenia posits that increased subcortical dopamine underpins psychosis. *In vivo* imaging studies indicate an increased presynaptic dopamine synthesis capacity in striatal terminals and cell bodies in the midbrain in schizophrenia; however, measures of the dopamine-synthesising enzyme, tyrosine hydroxylase (TH), have not identified consistent changes. We hypothesise that dopamine dysregulation in schizophrenia could result from changes in expression of dopamine synthesis enzymes, receptors, transporters or catabolic enzymes. Gene expression of 12 dopamine-related molecules was examined in post-mortem midbrain (28 antipsychotic-treated schizophrenia cases/29 controls) using quantitative PCR. TH and the synaptic dopamine transporter (DAT) proteins were examined in post-mortem midbrain (26 antipsychotic-treated schizophrenia cases per 27 controls) using immunoblotting. TH and aromatic acid decarboxylase (AADC) mRNA and TH protein were unchanged in the midbrain in schizophrenia compared with controls. Dopamine receptor D2 short, vesicular monoamine transporter (VMAT2) and DAT mRNAs were significantly decreased in schizophrenia, with no change in DRD3 mRNA, DRD3nf mRNA and DAT protein between diagnostic groups. However, DAT protein was significantly increased in putatively treatment-resistant cases of schizophrenia compared to putatively treatment-responsive cases. Midbrain monoamine oxidase A (MAOA) mRNA was increased, whereas MAOB and catechol-O-methyl transferase mRNAs were unchanged in schizophrenia. We conclude that, whereas some mRNA changes are consistent with increased dopamine action (decreased DAT mRNA), others suggest reduced dopamine action (increased MAOA mRNA) in the midbrain in schizophrenia. Here, we identify a molecular signature of dopamine dysregulation in the midbrain in schizophrenia that mainly includes gene expression changes of molecules involved in dopamine synthesis and in regulating the time course of dopamine action.

## Introduction

Subcortical dopamine dysregulation is considered the final common pathway in the pathophysiology of schizophrenia.^1, 2^ The dopamine hypothesis of schizophrenia was proposed, in part, based on the amelioration of symptoms by dopamine receptor D2 (DRD2) blockade, the main mechanism of action common to antipsychotic drugs.^3, 4^ A current version of the dopamine hypothesis posits that striatal hyperdopaminergia contributes to positive symptoms, and frontal cortical hypodopaminergia contributes to negative symptoms and cognitive deficits.^5^ The majority of dopamine neurons reside in the midbrain, including cell bodies in the substantia nigra and ventral tegmental area (VTA) that send projections to the striatum and cortex.^6^ Dopamine released in terminal fields and at dopamine cell bodies acts on dopamine receptors to initiate and regulate dopamine neurotransmission.^7^ Striatal hyperdopaminergia in schizophrenia has been attributed to over activity of the mesolimbic (ventral striatum) dopaminergic pathway; however, increased dopamine synthesis capacity is also found in the associative (dorsal) striatum^8^ and also likely involves increased dopamine synthesis capacity within the dopamine neurons. Two meta-analyses of 11 and 15 imaging studies, respectively, found significant elevations in dopamine synthesis capacity in the striatum in schizophrenia,^9, 10^ and a further imaging study identified increased dopamine synthesis capacity in the midbrain.^11^ Here, we ask whether molecular evidence of presynaptic dopamine changes can be found in the substantia nigra in people with schizophrenia.

Dopamine neurotransmission has multiple steps of possible regulation. Tyrosine hydroxylase (TH), the rate-limiting enzyme in dopamine biosynthesis, converts L-tyrosine to L-DOPA, which is then converted by aromatic acid decarboxylase (AADC) to dopamine.^7^ TH is regulated at transcriptional, translational and post-translational levels^12^ and, although in rodents electroconvulsive shock treatment does not change midbrain TH mRNA levels,^13^ regulation of midbrain TH mRNA does occur in response to pharmacological agents.^14, 15^ Studies measuring TH in the midbrain of people with schizophrenia do not concur, with no change,^16, 17^ increases^11, 18^ and region-specific decreases^17, 19^ reported in people with schizophrenia compared with controls. Although previously no change was found in AADC mRNA in the midbrain of schizophrenia patients,^16^ positron-emission tomography (PET) imaging studies have suggested increases in AADC activity in schizophrenia patients.^11^ Thus, further studies of TH and AADC within the basal ganglia are needed to determine whether molecular changes consistent with increased dopamine biosynthesis occur in schizophrenia.

Dopamine interacts with five G-protein-coupled receptors, dopamine receptor (DR) D2, DRD3 and DRD4 (inhibitory D2-like family) as well as DRD1 and DRD5 (excitatory D1-like family).^20^ DRD2 and DRD3 isoforms are expressed in the dopamine neuron somatodendritic field in the substantia nigra and VTA and at presynaptic terminals.^20, 21^ Presynaptic DRD2 and DRD3 full-length (referred to as DRD3) are autoreceptors, inhibiting dopamine neuron firing, dopamine release and terminating dopamine synthesis.^22, 23, 24, 25, 26^ Alternatively, spliced DRD3 variants that produce truncated proteins that do not bind dopamine have been identified, the most abundant being DRD3 non-functional (DRD3nf).^27, 28^ DRD3nf modulates dopamine reception by forming heteromers with DRD2 and DRD3, redirecting dopamine-binding receptors into an intracellular compartment and thus reducing DRD3 and DRD2 cell surface action.^29^ DRD3 mRNA is decreased, whereas DRD3nf mRNA is increased in the cortex (parietal, motor and anterior cingulate) of schizophrenia patients^27, 30^- setting a precedent for testing whether altered DRD3 expression occurs in the midbrain in schizophrenia.

We hypothesised that changes in mRNA encoding presynaptic dopamine receptors may contribute to changes in dopamine regulation in the midbrain in schizophrenia. A study of post-mortem midbrain showed increased DRD2 receptor binding in substantia nigra from schizophrenia cases.^31^ However, gene expression of DRD2 isoforms, DRD3 or DRD3nf within the midbrain in schizophrenia, has not yet been examined.

Dopamine action is terminated by reuptake from the synaptic cleft via the dopamine transporter (DAT) and dopamine is packaged into vesicles for release by vesicular monoamine transporter 2 (VMAT2),^32^ and these molecules also represent a possible site of dysregulation in schizophrenia. The ability of psychoactive drugs to bring about psychotic-like states by binding to DAT or VMAT2 (for review see Piccini^33^ and Chaudhry *et al.*^34^) imply that alterations in dopamine transport can contribute to psychosis. Dopamine is catabolised in dopamine neurons and glial cells by monoamine oxidases (MAOs) and catechol-O-methyl transferase (COMT).^7, 35, 36^ Midbrain monoamine oxidase A (MAOA) and MAOB are expressed in human substantia nigra,^37^ and inhibition of MAOs contributes to amphetamine-induced psychosis.^33^ Thus, decreased synthesis of dopamine breakdown or reduced dopamine transport proteins in schizophrenia may contribute to hyperdopaminergia or to dopamine dysregulation.

We ask whether gene expression of 12 dopamine-related molecules and/or protein expression of TH and DAT are changed in the substantia nigra from individuals who suffered from chronic schizophrenia compared with healthy individuals. We hypothesised that TH and AADC mRNA would be increased in the midbrain in schizophrenia with a corresponding increase in TH protein in the midbrain. Further, we hypothesised that DAT mRNA and protein would be decreased in the midbrain of people with schizophrenia compared with controls. We hypothesised that gene expression of other molecules involved in dopamine reception, transport or catabolism may contribute to dysregulation of dopamine neurotransmission, and thus may be changed in the midbrain in schizophrenia compared with controls.

## Materials and Methods

### Cohort tissue collection and demographic matching

**Figure 1:**
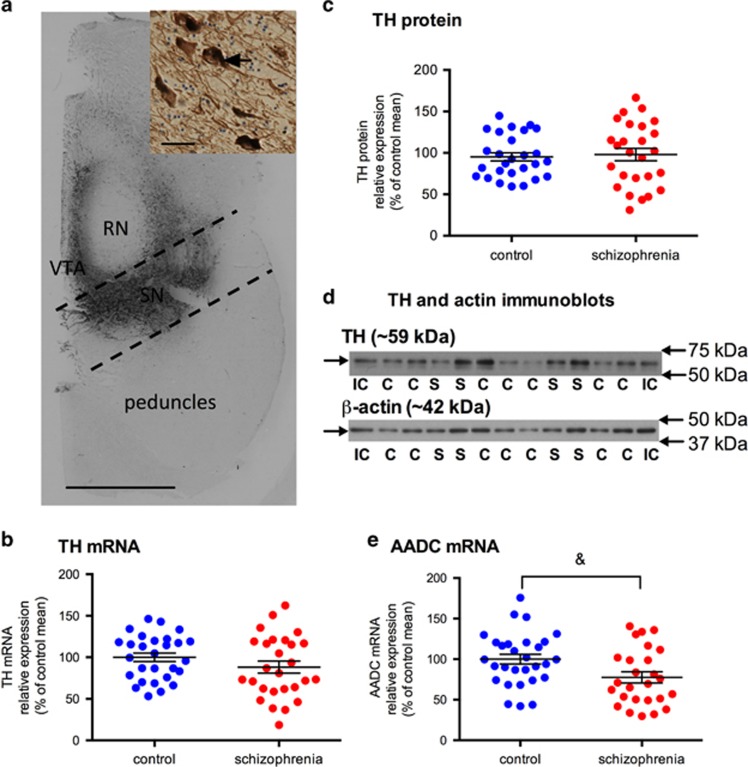
Dopamine synthesis enzymes, TH mRNA and protein and AADC mRNA, levels in the substantia nigra in control (blue circles) and schizophrenia cases (red circles). (**a**) TH immunohistochemistry in a human midbrain representative of our cohort. Dark brown staining is TH expression in cell bodies and processes. Dashed lines bound the area of tissue dissected and homogenised to enrich for midbrain dopamine neurons of the substantia nigra (black). Scale bar=1 cm. Inset shows TH-positive neurons with TH expression in the cytoplasm (arrow) and processes, scale bar=100 μm. (**b**) TH mRNA in the substantia nigra was not significantly different between control and schizophrenia cases (F=0.74; df=54; *P*=0.395). (**c**) TH protein in the substantia nigra was not significantly different between control and schizophrenia cases (F=0.304; df=53; *P*=0.584). (**d**) A single TH protein band (~59 kDa) and a single β-actin band (~42 kDa) were detected in all samples using immunoblotting. (IC, internal control; C control; S, schizophrenia). (**e**) AADC mRNA was decreased 22.49% in the substantia nigra in schizophrenia cases when compared with controls; however, this only reached a trend level (F=3.417; df=54; *P*=0.070). Data are mean±s.e.m., ^&^*P*<0.01. AADC, aromatic acid decarboxylase; RN, red nucleus; SN, substantia nigra; TH, tyrosine hydroxylase; VTA, ventral tegmental area.

Experiments involving human tissue were approved by the University of New South Wales Human Research Ethics Committee (HREC 12435). Hemisected fresh frozen midbrain tissue neuroanatomically matched at the level of the oculomotor nerve from 30 schizophrenia cases and 30 control individuals (14 μm sections mounted on gelatin-coated glass slides and adjacent 60 μm sections collected between wax paper) were provided by the New South Wales Brain Tissue Resource Centre. Sample size was selected based on previous post-mortem studies (minimum 25 cases needed to detected a 1.25-fold change, 80% power, *α*=0.05)^38^ and tissue availability. Substantia nigra was excised from midbrain cryostat-generated 60 μm slices based on TH immunolabelling of adjacent 14 μm slide-mounted sections (Figure 1a). Protein and mRNA were extracted from 6 × 60 μm midbrain slices each.

The final midbrain mRNA expression cohort comprised 28 schizophrenia cases and 29 controls (three cases excluded based on mRNA quality) and the final midbrain protein cohort comprised 26 schizophrenia cases and 27 controls (seven cases excluded because of unavailability of sufficient tissue or poor protein quality). In both cohorts, diagnostic groups were matched for age, gender and post-mortem interval (PMI), and in the mRNA cohort diagnostic groups were matched for RNA integrity number (RIN)^39^ (Table 1). All schizophrenia patients received antipsychotic medication. Throughout their illness 6–6 (mRNA–protein cohort) patients received first-generation antipsychotics only, 11–8 patients had predominantly first generation, 5–6 patients received equal first-generation and second-generation antipsychotics, 5–5 patients received predominantly second-generation antipsychotics and 1–1 received second generation only. As clozapine is generally only recommended after at least two trials of other antipsychotics have failed to have a benefit,^40^ clozapine treatment at time of death (*n*=7-7) was used as an indicator of possible treatment resistance versus all other antipsychotics at time of death (*n*=21–19). Antipsychotic medication was converted to chlorpromazine (CPZ) equivalents (lifetime, daily and last dose)^41, 42^ (Table 1). The schizophrenia cases were diagnosed with either more prevalent positive symptoms (*n*=22) or more prevalent negative symptoms (*n*=7) with one unknown. Toxicology screening of control cases at time of post-mortem revealed nothing (*n*=11), diazepam (*n*=1), cannabis (*n*=1), paracetamol (*n*=7), codeine (*n*=3) and blood pressure medication (*n*=2), and some (*n*=5) had no toxicology screen with cardiac failure recorded as cause of death. Toxicology screening of schizophrenia cases revealed no comorbid substance use (*n*=13), methadone and diazepam (*n*=1), diazepam and insulin (*n*=1), diazepam, pethidine, codeine and paracetamol (*n*=1), codeine, diazepam and morphine (*n*=1), paracetamol only (*n*=4), a non-steroidal anti-inflammatory (*n*=1), morphine (*n*=1) codeine (*n*=1) and ibuprofen (*n*=1) and screening not available (*n*=5). Alcohol consumption at time of death in controls and schizophrenia cases was as follows: nil (*n*=1–0), <20 g per day (*n*=17–22), 20–50 g per day (*n*=6–3), 50–80 g per day (*n*=1–0), >80 g per day (1–3) and unknown (*n*=4–2). A history of depression symptoms during lifetime was identified in eight schizophrenia cases (six were treated with serotonin-selective reuptake inhibitors (SSRIs) and two with tricyclic antidepressants) and in one control (treated with SSRI; Table 1).

### TH immunohistochemistry

Immunohistochemistry was performed as previously detailed.^43^ Frozen slides (14 μm) were thawed at room temperature and fixed in 4% paraformaldehyde in phosphate-buffered saline (pH 7.4, 10 min). After rinsing, endogenous peroxidase activity was blocked using 30% MeOH and 3% H_2_O_2_ (20 min), and slides were rinsed again and blocked with 10% normal horse serum (S2000, Vector Labs, Burlingame, CA, USA) for 1 h. Anti-TH mouse primary antibody was applied overnight at 4 °C at a 1:1000 dilution (MAB318, Merck Millipore, Bayswater, VIC, Australia). After rinsing, the slides were incubated with a horse anti-mouse IgG-biotinylated secondary antibody (BA-2000, Vector Labs) at a 1:500 dilution (1 h, room temperature) followed by rinsing and incubation with the avidin–biotin–peroxidase complex (PK4000, Vector Labs) and then application of 3,3′-diaminobenzidine solution (D5637, Sigma-Aldrich, St. Louis, MO, USA) to visualize TH immunoreactivity (2 min). The slides were rinsed, dehydrated, stained with Nissl (5 min exposure to 0.02% thionin) and cover-slipped. Slides were visualised on a Nikon Eclipse 80i light microscope. TH immunohistochemistry was scored (intensity: +, low; ++, medium; +++, high) based on overall TH staining intensity (including cell bodies and fibres) for the area of the midbrain equivalent to the area excised for homogenisation (indicated in Figure 1a), which includes both pars compacta and pars reticularis.^44^ These assessments were carried out blind to diagnosis by two raters with 87% concordance. Discordant ratings were re-assessed to determine the final rating.

### Quantitative real-time PCR analysis

Total RNA from substantia nigra samples was extracted with Trizol (Life Technologies, Scoresby, VIC, Australia). RNA quality was determined using the Agilent Bioanalyzer 2100 (Agilent Technologies, Santa Clara, CA, USA) and samples with low RIN were removed from gene expression analysis (one control and two schizophrenia cases). Complementary DNA was synthesised using the Superscript III First Strand Synthesis Kit (Life Technologies). Quantitative real-time PCR (qPCR) analysis was conducted as reported previously^38^ using the Applied Biosystems Prism 7900HT Fast Real-Time quantitative PCR system (Applied Biosystems, Life Technologies) and TaqMan gene expression assays (Applied Biosystems, Life Technologies). The following inventoried Taqman assays were used to measure the gene expression of three housekeeper genes (β-actin, Hs99999903; Tata-binding protein, Hs00427620; ubiquitin C, Hs00824723). The following inventoried TaqMan assays were used to measure gene expression of dopamine-related mRNAs: TH, Hs00165941; AADC, Hs01105048; MAOA, Hs00165140; MAOB, Hs00168533; COMT, Hs00241349; DAT, Hs00997364; VMAT2, Hs00996835; DRD2S, Hs01014210, DRD3, Hs00949496 and DRD3nf, Hs00945868. Custom probes were designed for DRD2L (AIHSPG8) and DRD2longer (AII1NNG) based on published sequences.^45^

Serial dilutions of pooled complementary DNA from all samples were included on every qPCR plate for quantitation of sample expression by the relative standard curve method. All qPCR reactions were performed in triplicate. The SDS 2.4 software (ABI, Life Technologies) was employed to analyse the qPCR data. Expression levels of the triplicate means of sample expression were normalised to the geomean of the housekeepers.^46^

### Protein extraction and western blotting

Western blotting was performed as previously described.^47^ Midbrain samples were homogenised (0.1 M Tris (pH 7.5), 50% glycerol, proteinase inhibitor cocktail and aprotinin (0.015 mM), all Sigma-Aldrich) using a handheld electric homogeniser (Polytron, Kinematica, Lucerne, Switzerland). Proteins were quantified using the Bradford protein assay (Sigma-Aldrich). An aliquot of each sample was pooled and run in duplicate on all gels for standardisation between immunoblots (internal control). Standard curves (0.5–15 μg protein) were run to determine the linear range of expression. Protein concentrations loaded for TH and DAT detection for two western runs/protein were 3 and 10 μg per sample for TH and DAT, respectively. Proteins were separated by SDS-PAGE (10% acrylamide) and transferred to nitrocellulose membranes (0.45 μm, Bio-Rad, Gladesville, NSW, Australia). Membranes were blocked in 5% non-fat milk (2 h, 4 °C) and incubated in primary antibody overnight at 4 °C. Primary antibodies were mouse anti-TH (1:5000; Merck Millipore, MAB318) and rabbit anti-DAT (1:200; Santa-Cruz, Dallas, TX, USA, sc-14002). Secondary antibodies were horseradish peroxidase-conjugated goat anti-mouse or goat anti-rabbit (both 1:2000; Merck Millipore). Bands were visualised using chemiluminescence (Amersham, GE Healthcare, Uppsala, Sweden) and captured on a Chemidoc XRS system (Bio-Rad). Band densities were determined using Quantity One Software (Bio-Rad, 4.6.3) and expressed numerically as quantity values (mm × intensity). All membranes were re-probed with mouse anti-β-actin (1:5000; Merck Millipore, MAB1501) as a loading control.

### Statistical analysis

Statistical tests were performed using SPSS (V23, IBM, Armonk, NY, USA). A *χ*^2^-test was used to determine the relationship between diagnosis and TH intensity scores determined from immunohistochemistry. The Grubb’s test (GraphPad Software, La Jolla, CA, USA) was used to exclude group outliers (0–2 individuals per group) in genes or proteins of interest after normalising to housekeepers. Independent-sample two-tailed *t*-tests were used to test for differences in mRNA and protein cohort demographics (age, PMI, pH and RIN (Table 1)) and individual housekeeper genes and the geomean of the housekeepers, as well as β-actin protein expression between control and schizophrenia cases.

The relative intensity of protein bands (TH, DAT and β-actin) was divided by the internal control for standardisation between blots. The relative intensity of TH and DAT bands was then normalised to the corresponding β-actin band relative intensity and tested for normality. To achieve a normal distribution, TH and DAT protein data underwent log10 transformations. The levels of the mRNAs of interest were normalised to the geometric mean (referred to as geomean3) of the housekeeper mRNAs (β-actin, Tata-binding protein and ubiquitin C) and the relative gene expression of each gene was tested for normality (Shapiro–Wilk test). Data that were not normally distributed were transformed. Monoamine oxidase and DRD3nf mRNA underwent log10 transformation and TH mRNA underwent a square root transformation.

If normality was not achieved after transformation, as in the case of COMT mRNA, the non-normally distributed mRNA data were analysed using the Mann–Whitney test. Spearman’s correlations for COMT mRNA were performed with continuous demographic variables (pH, PMI and RIN).

For all normally distributed mRNA and protein measures, Pearson’s correlations were performed with continuous demographic variables (pH, PMI, RIN (RNA cohort only)). For all mRNA and protein measures, Spearman’s correlations were conducted with antipsychotic drug measurements and duration of illness in the schizophrenia group. If a correlation was detected, analysis of covariance was used to test for diagnostic differences between controls and schizophrenia cases. Factorial analysis of variance was used to determine the effect of smoking status at time of death and agonal state on gene and protein expression in the diagnostic groups. If an effect was detected, this was included as a fixed factor in the analysis of covariance. If no correlations with demographic variables or effect of smoking or agonal state were detected, an independent sample two-tailed *t*-test was used to detect diagnostic differences.

Relative gene expression and relative protein expression data were converted to a percentage of the control group mean (that is, control mean was converted to 100%) and graphed as mean±s.e.m. Statistical significance was set at *P*⩽0.05.

The effect of antipsychotics on gene and protein expression was explored in three ways. First, correlation analyses of mRNAs and proteins with CPZ equivalents (described above) were performed. Second, comparisons between schizophrenia cases divided into those treated with mostly first-generation antipsychotics and those treated with mostly second-generation antipsychotics. Third, comparisons were made between cases using clozapine at time of death (possible treatment resistance) versus those on antipsychotics other than clozapine at time of death, although we acknowledge that not taking clozapine does not rule out treatment resistance. In addition, the effect of mostly positive or mostly negative symptoms and presence of depression symptoms during lifetime or no depression symptoms were explored in the schizophrenia cases. Student’s two-tailed *t*-tests were used to explore differences in gene or protein expression in the schizophrenia group based on antipsychotic treatment, symptoms and depression, and statistical significance was set at *P*⩽0.05. We highlight that this analysis is exploratory as the group sizes are modest and no corrections are made for multiple analyses.

## Results

### RNA and protein midbrain post-mortem cohort assessment of demographic variables

#### Relationship of demographic variables to each other and RIN

The relationships between demographic variables were assessed, and detailed statistical data are shown in Supplementary Material. Briefly, there were no correlations between age at death and brain pH in either the protein or RNA cohort or with RIN in the RNA cohort or based on diagnostic group in either cohort. As expected, RIN correlated positively with brain pH in the mRNA cohort, but RIN did not correlate with PMI. Brain pH and RIN did not vary significantly according to agonal state or smoking status at death in the whole cohort or when exploring diagnosis separately.

#### Correlation of demographic variables and housekeeper mRNAs and β-actin protein

The relationship between demographic variables and housekeeper mRNAs and β-actin protein were assessed. Detailed statistical analysis is reported in Supplementary Material. Briefly, none of the housekeeping genes, their geometric mean (geomean3) or β-actin protein expression varied between schizophrenia and controls. Age at death did not correlate with the expression of the housekeeper genes, geomean3 or with β-actin protein-relative intensity. As expected, brain pH was strongly positively correlated with geomean3 (*r*=0.458, *P*<0.0001, *n*=57) and gene expression of all three housekeepers individually. None of the housekeeper genes or the geomean3 correlated with PMI. Brain pH and PMI were not correlated with relative intensity of β-actin protein bands. All housekeeper genes and geomean3 (*r*=0.631; *P*<0.001; all *n*=57) showed strong positive correlations with RNA quality as determined by RIN, in the full cohort and by diagnostic group.

#### Correlations of genes/proteins of interest and post-mortem cohort demographic variables

Most genes of interest in the mRNA expression cohort positively correlated with both RIN and pH, except for DRD2longer, which only correlated with RIN and AADC, DRD3nf and DRD3 mRNAs, which only correlated with pH (Table 2). Only TH protein, not DAT protein levels correlated positively with pH (Table 2). No gene or protein of interest correlated with PMI, or age (Table 2). As such, pH and RIN or only RIN (DRD2longer), or only pH (AADC, DRD3, DRD3nf) were used as covariates to test for diagnostic differences in mRNA expression, and pH was used as a covariate when testing for diagnostic differences in TH protein expression. Smoking status at time of death had no effect on expression of any gene or protein of interest when exploring the full cohort or when based on diagnosis (Supplementary Table 1). No gene or protein of interest varied significantly according to agonal state in the whole cohort or when exploring diagnosis separately (Supplementary Table 1).

### TH mRNA levels, immunohistochemistry and protein levels

No significant difference in TH mRNA (F=0.74; df=54; *P*=0.395) levels in the substantia nigra between control and schizophrenia brains were identified by qPCR (Figure 1b). Visual inspection of TH immunohistochemistry confirmed that all sections were at a similar anatomical level of the midbrain (Figure 1a) and the quality of TH immunolabelling did not differ between midbrains from schizophrenia cases compared with control subjects. A *χ*^2^-test showed no relationship between the qualitative (+, ++, +++ rating) intensity of TH immunolabeling in the substantia nigra and diagnosis (*χ*^2^=1.59, df=2, *P*=0.24, *N*=54). A TH and β-actin band were detected at the expected molecular weights, ~59 and ~42 kDa, respectively, in all samples (Figure 1d), and no significant diagnostic difference in TH protein (F=0.304; df=53; *P*=0.584) was detected by western blotting (Figure 1c). Additionally, TH mRNA was positively correlated with TH protein levels within the midbrain (*r*=0.33, *N*=47, *P*=0.020).

### Aromatic acid decarboxylase mRNA levels

We found a decrease in AADC mRNA (22.49%) in substantia nigra from schizophrenia relative to controls; however, this difference did not reach statistical significance (F=3.417; df=54; *P*=0.070; Figure 1e).

### Dopamine D2 receptor splice variant mRNA levels

**Figure 2:**
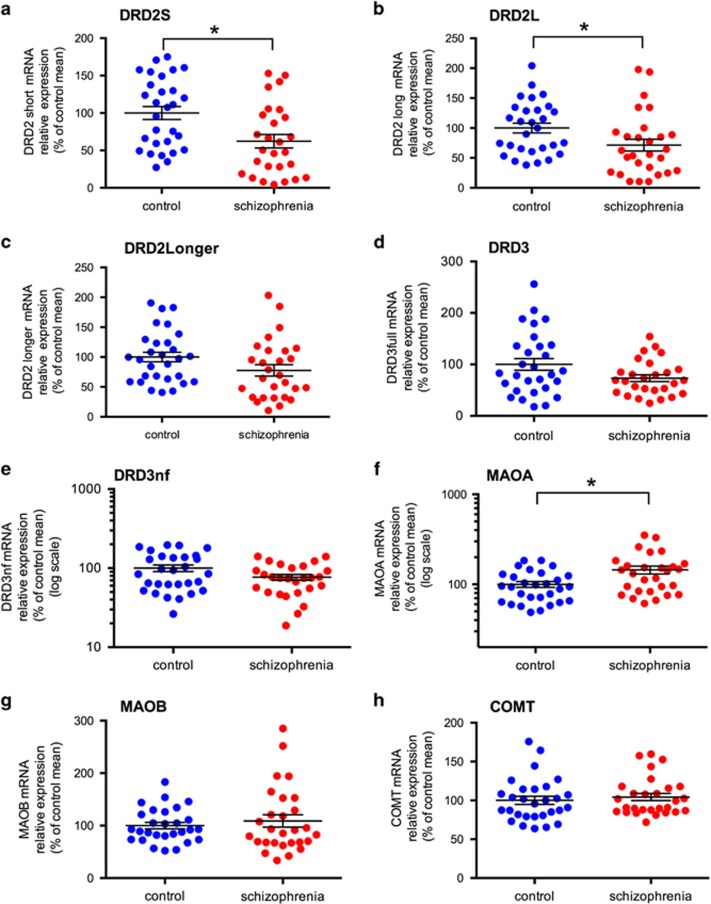
Dopamine receptor (DRD2S, DRD2L and DRD2Longer, DRD3, DRD3nf) and dopamine breakdown enzyme (MAOA, MAOB and COMT) gene expression in the substantia nigra in control (blue circles) and schizophrenia cases (red circles). (**a**) DRD2S mRNA was significantly decreased in substantia nigra from schizophrenia cases compared with control cases (F=3.05, df=55, *P*=0.018). (**b**) There was a significant decrease in expression of DRD2L mRNA in schizophrenia (F=3.98, df=56, *P*=0.051). (**c**) There was a trend for decreased expression for DRD2Longer mRNA (F=3.49, df=56, *P*=0.067). (**d**) DRD3 mRNA in the substantia nigra was not significantly different between control and schizophrenia cases (F=1.471; df=53; *P*=0.231). (**e**) DRD3nf mRNA in the substantia nigra was not significantly different between control and schizophrenia cases (F=0.949; df=55; *P*=0.334). (**f**) MAOA mRNA in the substantia nigra was significantly increased in schizophrenia cases compared with control cases (F=6.34; df=56; *P*=0.015). (**g**) MAOB mRNA in the substantia nigra was not significantly different between control and schizophrenia cases (F=0.81; df=53; *P*=0.371). (**h**) COMT mRNA in the substantia nigra was not significantly different between control and schizophrenia cases (*U*=462, *z*=0.89, *P*=0.371). Data are mean±s.e.m., *P⩽0.05. COMT, catechol-O-methyl transferase; DRD, dopamine receptor D; DRD3nf, DRD3 non-functional; MAOA, midbrain monoamine oxidase A.

DRD2 mRNA levels of all splice variants were reduced in the substantia nigra in schizophrenia compared with controls; DRD2S mRNA by 38% (F=3.05, df=55, *P*=0.018), DRD2L mRNA by 29% (F=3.98, df=56, *P*=0.051) and DRD2Longer mRNA by 22% (F=3.49, df=56, *P*=0.067; Figures 2a–c, respectively). Although the change in DRD2S mRNA was statistically significant and the change in DRD2L mRNA was at statistical significance, there was only a trend toward reduced DRD2longer mRNA in people with schizophrenia compared with controls.

### Dopamine D3 full-length and DRD3nf receptor mRNA levels

Although DRD3 was decreased by 26.76% and DRD3nf was decreased by 23.20% in substantia nigra of people with schizophrenia compared with controls, neither decrease reached statistical significance (F=1.471, df=53, *P*=0.231 and F=0.949, df=55, *P*=0.334, respectively; Figures 2d and e, respectively). There was also no statistical difference between the DRD3:DRD3nf ratio between control and schizophrenia cases (0.88±0.05 and 0.938±0.10; *t*=0,568, df=52, *P*=0.572).

### Gene expression of dopamine metabolic enzymes

MAOA mRNA was significantly increased by 45% in the substantia nigra in schizophrenia cases compared with controls (F=6.34, df=56, *P*=0.015), but no diagnostic changes were found in the levels of MAOB mRNA (F=0.81, df=53, *P*=0.372) or COMT mRNA (*U*=462, *z*=0.89, *P*=0.371; Figures 2f–h, respectively).

### Dopamine transporter mRNA and protein levels

**Figure 3:**
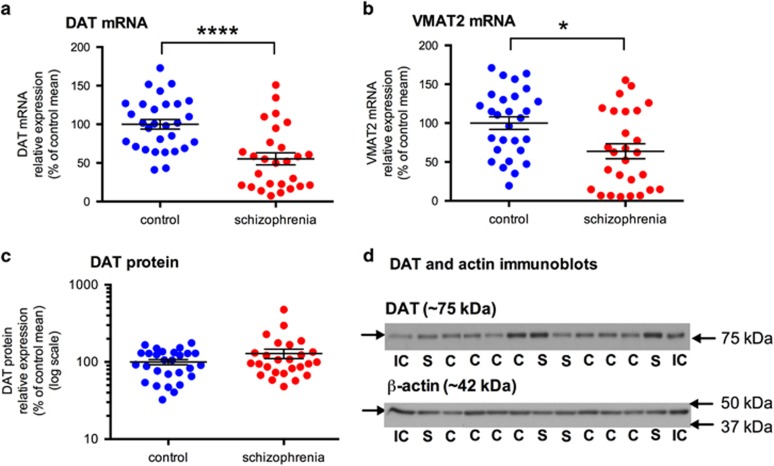
Gene expression of dopamine transporters, DAT and VMAT2, and DAT protein expression, in the substantia nigra in control (blue circles) and schizophrenia cases (red circles). (**a**) DAT mRNA levels were significantly decreased in schizophrenia (F=17.73; df=55; *P*<0.0001). (**b**) VMAT2 mRNA levels were significantly decreased in schizophrenia (F=6.54; df=54; *P*=0.014). (**c**) DAT protein in the substantia nigra was not significantly different between control and schizophrenia cases (*t*=−1.361; df=52; *P*=0.179). (**d**) Single DAT (~75 kDa) and β-actin bands (~42 kDa) were detected in all samples using immunoblotting. Data are mean±s.e.m.. *P⩽0.05, ****P⩽0.0001. C, control; DAT, dopamine transporter; IC, internal control; S, schizophrenia; VMAT2, vesicular monoamine transporter 2.

There was a highly significant 45% decrease in DAT mRNA levels (F=17.73; df=55; *P*<0.0001) and also a significant 37% decrease in the levels of VMAT2 mRNA (F=6.54; df=54; *P*=0.014) in the substantia nigra of schizophrenia cases when compared with controls (Figures 3a and b). A DAT protein band at the expected molecular weight (~75 kDa) was detected in all samples (Figure 3d). DAT mRNA and protein were not correlated (*r*=0.039, *N*=48, *P*=0.787). No significant difference in DAT protein expression was detected between control and schizophrenia (*t*=−1.361; df=52; *P*=0.179; Figure 3c).

#### Effect of antipsychotics and clinical state (depression over lifetime, symptoms) on gene and protein expression of dopamine-related molecules

None of the dopamine-related genes or proteins of interest measured in the substantia nigra from schizophrenia cases correlated significantly with measures of antipsychotic use or duration of illness from either the mRNA or protein expression cohorts (Table 2). In addition, there were no changes in any gene of interest or TH protein expression when those on clozapine at time of death (possible treatment-resistant schizophrenia) were compared with those treated with other antipsychotics (all; *t*<1.118, df=24–26, *P*>0.274). In contrast, we detected a 72.5% increase in DAT protein expression in cases on clozapine compared with cases treated with other antipsychotics (*t*<2.020, df=24, *P*=0.054). There were no changes in gene or protein expression when schizophrenia cases that received mostly first-generation antipsychotics were compared with schizophrenia cases that received mostly second-generation antipsychotics (all; *t*<0.787, df=24–26, *P*>0.243).

When schizophrenia cases displaying mostly positive or negative symptoms were compared, there was a significant 56.5% increase in DRD3 mRNA in the primarily positive symptoms group compared with those people with schizophrenia displaying mostly negative symptoms (*t*=−2.188, df=20, *P*=0.041). There were no changes in any other gene or protein expression based on symptoms. No gene or protein of interest varied significantly in the schizophrenia group based on the presence of depression symptoms within their lifetime (data not shown).

## Discussion

This is, to our knowledge, the first study to simultaneously examine the gene expression of 12 molecules with the potential to regulate dopamine neurotransmission within post-mortem midbrain from cases with schizophrenia compared with controls. This comprehensive analysis, utilising a demographically well-matched and characterised post-mortem midbrain cohort, has identified reductions in dopamine receptor and transporter mRNAs and increases in a catabolic enzyme mRNA. These significant and varied changes in multiple mRNAs in the midbrain of people with schizophrenia are not directly correlated with antipsychotic treatment estimates or illness duration and may not be readily explained by differences in smoking status or agonal state.

Whereas midbrain TH mRNA and protein levels were unchanged between schizophrenia and controls, we observed a greater range of values in schizophrenia compared with controls in both mRNA and protein, perhaps reflecting the contradictory evidence for changes (both increases and decreases) found in previous studies.^11, 16, 17, 18^ Although our study contributes to the evidence suggesting there are no overall changes in TH mRNA or protein levels in midbrain in schizophrenia, it cannot rule out a change in TH activity. Studies have shown that both posttranscriptional regulation of TH mRNA^48, 49^ and post-translational regulation of TH protein (e.g., phosphorylation)^50^ are important for TH level and activity. Midbrain TH mRNA and protein levels were highly correlated, thus suggesting that our measurements of human TH are accurate and valid. However, we measured only one TH mRNA transcript (pan) and four TH mRNA transcripts have been identified in human brain.^51, 52^ Thus, changes in individual TH transcripts and their corresponding protein isoforms may have been missed in our study. Additionally, our study did not compare subregions (VTA versus substantia nigra) or dorsal or ventral distribution within these regions and a recent study identified reductions in TH protein in subregions of the midbrain in schizophrenia.^19^

Increased activity of the second step in the dopamine synthesis pathway has been identified in schizophrenia. PET studies measuring 18F-DOPA uptake and conversion to 18F-dopamine show that the dopamine biosynthetic step that is AADC-dependent is elevated in people at ultrahigh risk for psychosis.^53^ Thus, increased dopamine synthesis capacity via AADC appears to be changed as a consequence of schizophrenia and may not be only secondary to antipsychotic treatment. In contrast, PET imaging studies in schizophrenia patients, who were antipsychotic-naive or had not received treatment in the previous 6 months and subsequently treated subchronically (20–45 days) with haloperidol (a first-generation antipsychotic with high affinity for DRD2), showed a downregulation of this AADC-dependent step.^54^ Contrary to our hypothesis, but in line with the study by Grunder *et al.,*^54^ we found a trend for a decrease (22.9%) in AADC mRNA levels in the midbrain of patients with schizophrenia that suggests that there could be less synthesis of AADC in chronic, medicated patients. In rodent whole-brain homogenates, haloperidol and loxapine treatments increased AADC mRNA, whereas the second-generation antipsychotic sulpiride (a DRD2 and DRD3 antagonist) did not.^55^ The differential effects of distinct antipsychotics on AADC gene expression may reduce the ability to detect a consistent change in AADC in post-mortem tissue and exposure to antipsychotics may mask potential changes in AADC in schizophrenia. It is proposed that some schizophrenia patients do not respond to antipsychotic treatment as they do not exhibit the elevation in dopamine synthesis capacity that is typically associated with the disorder.^56^ However, using clozapine at the time of death as a proxy for possible treatment resistance^40^ did not reveal a difference in AADC gene expression. Interestingly, there was an indication of a negative correlation with the last CPZ equivalent dose and AADC mRNA, suggesting that in our study, the AADC mRNA reductions may be due to antipsychotic treatment.

As a major finding of this study, we found a 45% decrease in DAT mRNA in schizophrenia. This may seem at odds with our lack of ability to detect a change in DAT protein levels in the substantia nigra in schizophrenia. The lack of diagnostic change in DAT protein could suggest that DAT mRNA changes may not have an impact on protein levels in midbrain or that the protein function/stability may be altered causing feedback changes on DAT transcription. Alternatively, there may be increased translation of DAT protein or decreased DAT utilisation and breakdown, either of which could result in no change in steady-state DAT protein levels with a decrease in DAT mRNA. However, we report a decrease in DAT protein in cases treated with antipsychotics other than clozapine (that is, potentially treatment-responsive) relative to those treated with clozapine (potentially treatment-resistant)—such differences may contribute to the difficulty in detecting a diagnostic change in DAT protein expression overall and suggest that posttranscriptional regulation of DAT may vary with clinical state. Alternatively, it is possible that the decrease in DAT mRNA may be reflected in DAT protein changes in either the dorsal or ventral striatum or in cortical sites, which were not examined in this study. As dopamine action in the striatum is primarily terminated by dopamine reuptake from the synaptic cleft by DAT, less DAT levels or DAT action leading to slower termination of dopamine neurotransmission could contribute to hyperdopaminergia in schizophrenia. In the striatum, post-mortem studies report reduced levels of DAT binding in schizophrenia;^57, 58^ however, imaging studies indicated no change in striatal DAT binding in schizophrenia patients.^9, 59, 60^ Similar binding studies have not been carried out in the midbrain of schizophrenia patients in whom dopamine can be released in the somatodendritic field to regulate dopamine neuron activity via feedback inhibition. Further work including study of DAT protein, binding and activity, both in post-mortem and PET imaging studies in the brains of people with schizophrenia (ideally in both medicated and antipsychotic naïve patients and taking into account clinical state such as treatment resistance) compared with controls, are needed to more fully characterize the anatomical sites of DAT abnormalities, especially in the midbrain, and to better understand the implications of our current findings. This is, however, to our knowledge, the first study to implicate reduced midbrain DAT gene expression in the pathophysiology of schizophrenia, highlighting a putative major dysregulation of DAT.

In addition to a reduction in the mRNA encoding the main dopamine transporter localized to the outer cell membrane, we also find reduced mRNA encoding dopamine transporter localized to the vesicles (that is, VMAT2) in schizophrenia, VMAT2 mRNA does not appear to be regulated by antipsychotics in our study or in others.^61^ A decrease in VMAT2 gene expression in the substantia nigra may contribute to reduced VMAT2 protein and suggests less efficient vesicular packaging of dopamine; however, less VMAT2 action can contribute to hyperdopaminergia in certain contexts as inhibition of VMAT2 contributes to amphetamine-induced psychosis.^34^ In contrast to our study, VMAT2 binding was found to be increased in the ventral brainstem in a PET imaging study in schizophrenia;^61^ however, this apparent difference may be due to regional differences in the expression of dopamine molecules in the brainstem. These observations set the stage for designing experimental systems that would mimic the putative state of transporter abnormalities found in schizophrenia and could serve as a platform for testing the impact of novel therapies.

In addition to changes in dopamine synthesis, transporter and receptor mRNAs, we found changes in mRNA for one catabolic enzyme, MAOA, that can also terminate dopamine action. If there is an increase in dopamine in the synaptic cleft in people with schizophrenia, we suggest that it is possible that the increase in MAOA mRNA may reflect a compensatory response to balance extracellular dopamine levels. However, MAOA is also involved in the breakdown of other neurotransmitters including serotonin and norepinephrine, and MAOA variants are implicated in psychiatric disorders.^62^ Serotonin released in the substantia nigra by dorsal raphe serotonergic fibres results in activation of 5-HT2 receptors and subsequent inhibition of dopamine neuron firing.^63^ Therefore, more MAOA synthesis/activity in the midbrain in schizophrenia could lead to increased serotonin breakdown and reduced inhibitory modulation of dopamine neurons by serotonin, thus contributing to the dopamine dysregulation. Increased cortical COMT activity, inferred from genetic changes, was linked to increased midbrain TH mRNA in normal subjects;^64^ however, we find no change in midbrain COMT mRNA levels in people with schizophrenia compared with controls, supporting previous studies in other brain regions finding that changes in COMT mRNA levels do not appear to be a predominant mechanism associated with the pathophysiology of schizophrenia.^64, 65, 66, 67^

We find decreased DRD2S mRNA in the substania nigra in schizophrenia in our cohort and DRD2S mRNA levels do not correlate with antipsychotic measurements. This supports previous studies showing that treatment with antipsychotics did not change DRD2 mRNA in rodent cortex or striatum.^68^ Our gene expression findings are in contrast to a previous post-mortem study showing increased DRD2 receptor binding in midbrain homogenates from drug-naive and drug-treated schizophrenia cases compared with controls;^31^ thus, we speculate that the changes we find in DRD2 mRNA may result in less DRD2 protein in the presynaptic terminal rather than in the midbrain. The binding of dopamine to the DRD2S autoreceptor on the presynaptic terminal results in the inhibition of dopamine synthesis and release and, thus, a decrease in dopamine neurotransmission.^24, 25^ We speculate that if the decrease in DRD2S gene expression is reflected in less DRD2S protein expression in the terminal field of the striatum in schizophrenia, this could contribute to an increase in striatal dopamine neurotransmission. In contrast to our evidence of decreased DRD2, we and others, find increased DRD2S mRNA in DLPFC in schizophrenia,^45, 69^ indicating potential region-specific alterations of this receptor splice variant mRNA in schizophrenia. Although DRD2L and DRD2longer are traditionally thought of as postsynaptic receptors found at terminal sites, we have readily measured mRNA of both of these isoforms in the human midbrain and report decreases in these isoforms in schizophrenia. This suggests that these potential decreases in DRD2 isoforms (short and long) may contribute to presynaptic or dendritic pathophysiology in schizophrenia. Although we did not identify a diagnostic difference in DRD3 or DRD3nf mRNA, schizophrenia cases exhibiting more positive symptoms had a 56% increase in DRD3 mRNA compared with those with more negative symptoms. Thus, increased DRD3 mRNA and possibly protein may also contribute to dopamine dysregulation in some people with schizophrenia.

**Figure 4:**
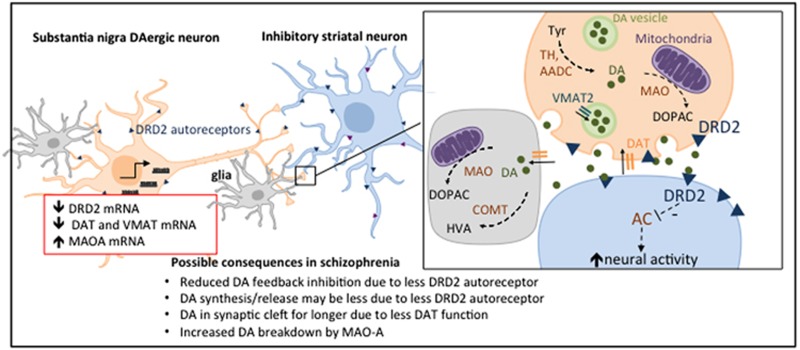
Proposed model of dopamine dysregulation in the nigrostriatal pathway in schizophrenia. Gene expression of multiple DA-regulating molecules (red box) involved in autoreception (DRD2), transport (DAT and VMAT) and catabolism (MAO) are changed in the substantia nigra in schizophrenia, and together may contribute to DA dysregulation at the level of the DA cell bodies and/or at the DA terminals in the striatum. DA is synthesised from tyrosine (tyr) by TH and AADC, but these do not appear to be changed in level. MAO and COMT breakdown DA to metabolites, DOPAC and HVA and MAO mRNA is increased. DAT removes DA from the synaptic cleft and is reduced at the mRNA level but not at the protein level in the midbrain. VMAT packages DA into vesicles and is reduced at the mRNA level. DRD2 (blue triangle) autoreception inhibits DA firing, DA synthesis and DA release, and we found a significant decrease in DRD2S mRNA. DRD3 mRNA was unchanged. DA activation of DRD2 (blue triangle) on the postsynaptic neuron inhibits AC leading to disinhibition of the inhibitory medium spiny neurons. Increased DRD2 expression in the striatum in schizophrenia^69^ would further contribute to DA dysregulation. AADC, aromatic acid decarboxylase; AC, adenylate cyclase; COMT, catechol-O-methyl transferase; DA, dopamine; DRD, dopamine receptor D; MAO, monoamine oxidase; TH, tyrosine hydroxylase; VMAT2, vesicular monoamine transporter 2.

In sum, we find evidence of changes in gene expression of multiple molecules that may act individually or in combination to contribute to the dopaminergic system dysregulation identified in people with schizophrenia (see Figure 4 for an integrative schematic of putative changes). Further support for actual changes in dopamine content, release or bioactivity and/or activity of biosynthetic/catabolic enzymes in schizophrenia brain is needed to more fully interpret these transcriptional changes. The differences reported here could contribute to a hyperdopaminergic state or may signify compensatory mechanisms for a hypodopaminergic state, rather than a causal process. It is noteworthy that there is greater variability in some gene expression levels in the midbrain from schizophrenia cases than in the control cases, and this may reflect the known heterogeneity of the disorder. Gene expression only provides clues as to whether protein levels may be changed and cannot ascertain if differences in protein levels may also exist in the brain of people with schizophrenia; therefore, our findings of changes in VMAT, MAOA and DRD2S should be extended to include protein measurements. However, when we did measure DAT protein levels, the changes did not directly reflect our mRNA levels. Proteins are regulated by post-translational mechanisms and so levels of protein as detected by western blot analysis do not reflect protein activity. We also do not know from gene expression data in the cell bodies at which location within the dopaminergic neurons—terminals, dendrites and cell bodies—these gene expression changes may be reflected in protein changes. A post-mortem study only allows one time point to be evaluated, and we are measuring gene and protein expression following severe chronic illness of different durations and prolonged treatment with different antipsychotics.^39^ However, these studies provide a basis for interpreting changes seen in *in vivo* studies in schizophrenia patients and the putative clues they provide to pathophysiology, and potential therapeutic targets beyond DRD2 antagonism can be followed up in preclinical models of dopamine dysregulation.

We conclude that, in a demographically well-matched and characterised post-mortem midbrain cohort, gene expression of multiple dopamine-regulating molecules are changed in the substantia nigra in schizophrenia and that changes in autoreception, transport and possible catabolism together may contribute to the dopamine dysregulation in schizophrenia (Figure 4). This study begins to address a vital knowledge gap in the literature with respect to the molecular nature of dopamine dysfunction within the dopamine neurons of people with schizophrenia, and extends the evidence of a presynaptic dysfunction from dopamine synapses in the striatum to possible changes in cell bodies in the midbrain and also to the implication of multiple dopamine-regulating loci—all potential targets for the development of novel therapeutics that may require testing in complex settings of dopamine dysregulation.

## Figures and Tables

**Table 1:** Demographic details of post-mortem midbrain mRNA and protein cohorts

*Demographic*	*mRNA cohort*			*Protein cohort*		
	*Control mRNA (*n=*29)*	*Schizophrenia mRNA (*n=*28)*	*Statistics mRNA*	*Control protein (*n=*27)*	*Schizophrenia protein (*n=*26)*	*Statistics protein*
Age (years)	51.2 (22–69)	51.4 (26–67)	*P*=0.10, df=55, *t*=−0.06	52.21 (22–69)	52.29 (26–67)	*P*=0.82, df=52, *t*=−0.23
Gender (M, F)	(20, 9)	(19, 9)	—	(19, 8)	(16, 10)	—
pH	6.67±0.26	6.51±0.20*	*P*=0.01, df=55, *t*=2.6	6.69±0.24	6.51±0.23*	*P*=0.01, df=52, *t*=2.6
PMI (h)	31.9±10.12 (15–50)	35.7±17.71 (5–72)	*P*=0.33, df=55, *t*=−0.98	32.75±9.90 (15–50)	38.21±18.20 (5–72)	*P*=0.17, df=52, *t*=−1.38
RIN	5.6±1.14	5.6±1.31	*P*=0.95, df=55, *t*=−0.06	na	na	na
Duration of illness (year)	na	28.31±12.72 (4–49) (28.19–39)	—	na	29.12±13.02 (4–49) (30, 19.3–39)	—
Daily chlorpromazine equivalent dose (mg)	na	736.45±520.50 (*n*=22) (583.0, 387.5–825.0)	—	na	716.37±557.76 (*n*=19) (553.0, 350.0–784.0)	—
Last-recorded chlorpromazine equivalent dose (mg)	na	597.54±497.64 (*n*=28) (462.5, 192.5–927.0)	—	na	616.20±506.60 (*n*=26) (475.0, 250.0–1025.0)	—
Life time chlorpromazine equivalent dose (g)	na	8231.44±8714.24 (*n*=22) (4726.8, 3442.0–8563.0)	—	na	8427.92±9348.47 (*n*=19) (4471.3, 3036.8–8212.5)	—
Depression symptoms over lifetime (yes, no, unknown)	1 (SSRI), 26, 2	8 (6 on SSRIs, 2 on TCAs), 19, 1	—	1 (SSRI), 24, 2	7 (5 on SSRIs, 2 on TCAs), 18, 1	—
Symptoms (mostly positive, mostly negative, unknown)	na	18, 7, 3	—	na	19, 7, 0	—
Agonal state (optimal/good/poor/unknown)	18/10/0/1	17/9/0/2	—	17/10/0/0	15/10/0/1	—
Smoker at time of death (yes/no/unknown)	11/12/6	16/6/6	—	11/11/5	16/6/4	—

Abbreviations: F, female; M, male; na, not applicable; PMI, post-mortem interval; RIN, RNA integrity; SSRI, selective serotonin reuptake inhibitor; TCA, tricyclic antidepressant.

Data are mean±s.d. (range) (median, interquartile range). *P⩽0.05.

**Table 2:** Correlations between dopamine-related gene or protein expression RIN, pH, PMI, age at death, measures of antipsychotic drug treatment and duration of illness

*Gene/protein of interest*	*RIN, PMI, pH, age*	N	P	*Correlation coefficient*	*Chlorpromazine equivalent*	N	P	*Correlation coefficient*
TH mRNA	RIN	55	0.001	0.457*	Life time	21	0.987	0.004
	pH	55	0.004	0.378*	Mean daily	21	0.913	0.025
	PMI	55	0.716	−0.5	Last dose	27	0.365	−0.181
	Age	55	0.903	0.17	Illness duration	27	0.824	0.045
AADC mRNA	RIN	55	0.216	0.113	Life time	21	0.575	−0.13
	pH	55	0.03	0.292*	Mean daily	21	0.693	−0.092
	PMI	55	0.839	−0.028	Last dose	26	0.165	−0.281
	Age	55	0.288	0.146	Illness duration	26	0.627	0.1
DAT mRNA	RIN	56	0.017	0.319*	Life time	21	0.239	−0.269
	pH	56	0.009	0.344*	Mean daily	21	0.315	−0.231
	PMI	56	0.6	−0.072	Last dose	27	0.198	−0.256
	Age	56	0.924	−0.013	Illness duration	27	0.886	0.029
VMAT2 mRNA	RIN	55	0.001	0.514*	Life time	21	0.61	−0.118
	pH	55	0.001	0.485*	Mean daily	21	0.689	−0.093
	PMI	55	0.933	0.012	Last dose	27	0.376	−0.177
	Age	55	0.354	0.127	Illness duration	27	0.702	0.077
MAOA mRNA	RIN	57	0.001	−0.528*	Life time	22	0.644	0.104
	pH	57	0.001	−0.498*	Mean daily	22	0.793	0.059
	PMI	57	0.238	0.159	Last dose	28	0.408	−0.163
	Age	57	0.64	0.063	Illness duration	28	0.403	0.164
MAOB mRNA	RIN	54	0.007	−0.366*	Life time	20	0.677	−0.099
	pH	54	0.014	0.332*	Mean daily	20	0.789	−0.064
	PMI	54	0.526	0.088	Last dose	26	0.482	−0.144
	Age	54	0.236	0.164	Illness duration	26	0.559	0.12
COMT mRNA	RIN	57	0.001	−0.457*	Life time	22	0.687	−0.091
	pH	57	0.001	−0.518*	Mean daily	22	0.32	−0.222
	PMI	57	0.729	0.047	Last dose	28	0.771	0.058
	Age	57	0.355	−0.125	Illness duration	28	0.888	0.028
DRD2short mRNA	RIN	56	0.001	0.441*	Life time	21	0.61	−0.118
	pH	56	0.001	0.493*	Mean daily	21	0.445	−0.176
	PMI	56	0.894	0.018	Last dose	27	0.529	−0.127
	Age	56	0.432	0.107	Illness duration	27	0.698	0.078
DRD2L mRNA	RIN	57	0.001	0.415*	Life time	22	0.32	−0.223
	pH	57	0.01	0.340*	Mean daily	22	0.131	−0.332
	PMI	57	0.705	0.051	Last dose	28	0.417	−0.16
	Age	57	0.4	0.114	Illness duration	28	0.929	0.018
DRD2longer mRNA	RIN	57	0.052	0.259*	Life time	22	0.582	−0.124
	pH	57	0.09	0.227&	Mean daily	22	0.303	−0.23
	PMI	57	0.431	0.106	Last dose	28	0.256	−0.222
	Age	57	0.4	0.114	Illness duration	28	0.991	−0.002
DRD3 mRNA	RIN	54	0.254	0.158	Life time	20	0.613	−0.12
	pH	54	0.011	0.344*	Mean daily	20	0.104	−0.375
	PMI	54	0.49	0.096	Last dose	26	0.444	0.157
	Age	54	0.35	−0.13	Illness duration	26	0.368	−0.184
DRD3nf mRNA	RIN	56	0.206	0.172	Life time	21	0.407	−0.191
	pH	56	0.031	0.288*	Mean daily	21	0.125	−0.346
	PMI	56	0.165	0.188	Last dose	27	0.842	0.04
	Age	56	0.275	−0.148	Illness duration	27	0.173	−0.27
TH protein	pH	54	0.002	0.413*	Life time	20	0.349	−0.221
	PMI	54	0.614	−0.07	Mean daily	20	0.992	0.002
	Age	54	0.95	−0.009	Last dose	26	0.602	0.107
					Illness duration	25	0.403	0.175
DAT protein	pH	54	0.875	0.022	Life time	20	0.724	0.084
	PMI	54	0.476	0.099	Mean daily	20	0.615	0.12
	Age	54	0.188	−0.182	Last dose	26	0.797	0.053
					Illness duration	25	0.736	−0.071

Abbreviations: AADC, aromatic acid decarboxylase; COMT, catechol-O-methyl transferase; DAT, dopamine transporter; DRD, dopamine receptor D; DRD3nf, DRD3 non-functional; MAOA, midbrain monoamine oxidase A; PMI, post-mortem interval; RIN, RNA integrity number; TH, tyrosine hydroxylase; VMAT2, vesicular monoamine transporter 2. *P⩽0.05.
