# Supplementary material for: Putative presynaptic dopamine dysregulation in schizophrenia is supported by molecular evidence from post-mortem human midbrain
Source: Transl Psychiatry. 2017 Jan 17;7(1):e1003–. doi: 10.1038/tp.2016.257 (PMC5545725; doi:10.1038/tp.2016.257)
Supplement: Supplementary Information [file tp2016257x1.docx]

**Supplementary data**

*RNA and protein midbrain post-mortem cohort: detailed assessment of demographic variables*

Relationship of demographic variables to each other and RIN.

**Age at Death**

Age at death was not correlated with brain pH or RIN in the full mRNA cohort (n=57, r=-0.059 and -0.087, *p*=0.665 and p=0.519, respectively) or in either diagnostic group (control pH, RIN: n=29, r=0.086 and -0.020, *p*=0.659 and 0.916; schizophrenia pH, RIN: n=28, r=-0.245 and -0.126, p=0.209 and 0.524). Age at death was not correlated with brain pH of the full protein cohort (n=54, r=-0.179, *p*=0.195). When exploring the diagnostic groups separately, age at death was not correlated with brain pH in the control group (n=28, r=0.025, *p*=0.9) but was negatively correlated with brain pH in the schizophrenia group (n=26, r=-0.455, *p*=0.020).

**Smoking at death**

Smoking at death (Supplementary Table 1) did not relate to brain pH or RIN in the full mRNA cohort or pH in the protein cohort (all: df=1, F<0.290, all *p*>0.571) or when split on the basis of diagnosis (diagnosis*smoking at death pH: all df=1,44, F<1.70, all *p*>0.20). RIN correlated positively with brain pH (n=57, r=0.355, *p*=0.007) in the mRNA cohort, but RIN did not correlate with PMI (n=57, r=-0.198, *p*=0.140).

Correlation of demographic variables and housekeeper mRNAs and β-actin protein

None of the housekeeping genes, nor their geometric mean (geomean3), varied between schizophrenia and controls (β-actin: t=0.956, df=55, *p*=0.343; TBP: t = 1.003, df = 55, p = 0.320; UBC: t=0.194, df=55, *p*=0.847; geomean3: t=0.745, df=55, *p*=0.459). β-actin protein expression did not differ between schizophrenia and control groups for either protein of interest (TH: t=-0.108, df=52, *p*=0.914; DAT: t=0.034, df=52, *p*=0.973).

**Age at death**

Age at death did not correlate with the expression of the housekeeper genes or with geomean3 (β-actin: r=-0.206, *p*=0.123; TBP: r=-0.114, *p*=0.397; UBC: r=-0.111, *p*=0.413; geomean3: r=-0.151, *p*=0.263; all n=57). There were also no correlations between age at death and expression of housekeepers and geomean3 when diagnostic groups were explored separately (data not shown). Age at death did not correlate with the relative intensity of β-actin protein bands from either the TH or DAT immunoblots (TH: r=-0.065, *p*=0.640; DAT: r=0.120, *p*=0.387; all n=54). There were also no correlations between age at death and relative intensity of β-actin protein bands when diagnostic groups were explored separately (data not shown).

**pH and PMI**

Brain pH was strongly positively correlated with geomean3 and gene expression of all three housekeepers (geomean3: r=0.458, *p*<0.0001; β-actin: r=0.386, *p*=0.003; TBP: r=0.434, *p*=0.001; UBC r=0.517, *p*<0.001; all n=57). Positive correlations between pH and gene expression of the housekeepers and geomean3 were present when exploring diagnostic groups (all r>0.4, *p*<0.05, n=29/28 control/schizophrenia), except for TBP mRNA in the schizophrenia group (n=28, r=0.351, *p*=0.067) and β-actin mRNA in the control group (n=29, r=0.328, *p*=0.082) where the positive correlations reached trend level. Brain pH was not correlated with relative intensity of β-actin protein bands (TH: r=0.149, *p*=0.282; DAT: r=0.173, *p*=0.212; all n=54), nor when exploring diagnostic groups (data not shown). The relative intensity of β-actin bands did not correlate with PMI (TH: r=-0.061, *p*=0.659; DAT: r=0.153, *p*=0.269; all n=54), nor when exploring diagnostic groups (data not shown). We did not detect a relationship between agonal state and brain pH or RIN in either the mRNA or protein cohort, or when diagnostic groups were individually examined (all p>0.30)

**RIN and PMI**

All housekeeper genes and geomean3 showed strong positive correlations with RNA quality as determined by RIN (β-actin: r=0.547, *p*<0.001; TBP: r=0.649, p<0.001; UBC: r=0.628, p<0.001; geomean3: r=0.631; p<0.001; all n=57). All of the housekeeper genes and the geomoean3 in both the control and schizophrenia groups showed strong positive correlations to RIN (control all: r>0.40, p<0.05; schizophrenia all: r>0.70, p<0.001). None of the housekeeper genes or the geomean3 correlated with PMI (all r<-0.11; p>0.5; n=57). When exploring diagnostic groups, PMI did not correlate with any housekeeper gene or the geomean3 (control all: r<0.10; p>0.7, schizophrenia all r<0.17; p>0.4) and PMI did not correlate with β-actin protein from TH or DAT immunoblots (data not shown).

Supplementary Table 1: Effect of agonal state and smoking status on dopamine-related gene or protein expression depending on diagnosis.

| Gene of interest | Agonal state  smoking | df | *p* | F |
| --- | --- | --- | --- | --- |
| TH mRNA | Agonal state  Agonal state*diagnosis  Smoking  Smoking*diagnosis | 1  1,52  1  1,42 | 0.519  0.123  0.391  0.728 | 0.422  2.465  0.752  0.123 |
| AADC mRNA | Agonal state  Agonal state*diagnosis  Smoking  Smoking*diagnosis | 1  1,52  1  1,43 | 0.118  0.296  0.628  0.089 | 2.533  1.115  0.438  3.043 |
| DAT mRNA | Agonal state  Agonal state*diagnosis  Smoking  Smoking*diagnosis | 1  1,53  1  1,43 | 0.102  0.835  0.980  0.350 | 2.770  0.044  0.001  0.874 |
| VMAT2 mRNA | Agonal state  Agonal state*diagnosis  Smoking  Smoking*diagnosis | 1  1,52  1  1,42 | 0.824  0.205  0.084  0.412 | 3.116  0.412  0.050  1.663 |
| MAOA mRNA | Agonal state  Agonal state*diagnosis  Smoking  Smoking*diagnosis | 1  1,53  1  1,44 | 0.942  0.716  0.287  0.487 | 0.005  0.134  1.163  0.493 |
| MAOB mRNA | Agonal state  Agonal state*diagnosis  Smoking  Smoking*diagnosis | 1  1,51  1  1,41 | 0.499  0.683  0.634  0.554 | 0.465  0.168  0.230  0.356 |
| COMT mRNA | Agonal state  Agonal state*diagnosis  Smoking  Smoking*diagnosis | 1  1,53  1  1,44 | 0.834  0.951  0.573  0.997 | 0.040  0.004  0.323  0.001 |
| DRD2short mRNA | Agonal state  Agonal state*diagnosis  Smoking  Smoking*diagnosis | 1  1,53  1  1,43 | 0.019  0.116  0.960  0.368 | 5.892*  0.734  0.003  0.830 |
| DRD2L mRNA | Agonal state  Agonal state*diagnosis  Smoking  Smoking*diagnosis | 1  1,53  1  1,44 | 0.056  0.316  0.845  0.657 | 3.830&  1.027  0.039  0.200 |
| DRD2longer mRNA | Agonal state  Agonal state*diagnosis  Smoking  Smoking*diagnosis | 1  1,53  1  1,44 | 0.166  0.668  0.779  0.390 | 0.194  0.187  0.080  0.731 |
| DRD3 mRNA | Agonal state  Agonal state*diagnosis  Smoking  Smoking*diagnosis | 1  1,50  1  1,41 | 0.645  0.638  0.328  1.951 | 0.215  0.224  0.984  0.171 |
| DRD3nf mRNA | Agonal state  Agonal state*diagnosis  Smoking  Smoking*diagnosis | 1  1,52  1  1,42 | 0.575  0.754  0.267  0.147 | 0.319  0.099  1.269  0.704 |
| TH protein | Agonal state  Agonal state*diagnosis  Smoking  Smoking*diagnosis | 1  1,49  1  1,43 | 0.251  0.111  0.772  0.953 | 0.619  2.641  0.085  0.003 |
| DAT protein | Agonal state  Agonal state*diagnosis  Smoking  Smoking*diagnosis | 1  1,49  1  1,43 | 0.361  0.253  0.321  0.430 | 0.850  1.342  1.010  0.637 |
